# Social distancing cut down the prevalence of acute otitis media in children

**DOI:** 10.3389/fpubh.2023.1079263

**Published:** 2023-01-27

**Authors:** Huiqian Yu, Dantong Gu, Fangzhou Yu, Qingzhong Li

**Affiliations:** State Key Laboratory of Medical Neurobiology, NHC Key Laboratory of Hearing Medicine Research, Department of ENT Institute and Otorhinolaryngology, Eye and ENT Hospital, Fudan University, Shanghai, China

**Keywords:** social distancing, acute otitis media, epidemiology, children, COVID-19

## Abstract

**Objectives:**

To evaluate the additional, unintended benefits of social distancing in cutting down the prevalence of acute otitis media (AOM) in children, especially during coronavirus disease 2019 (COVID-19) periods.

**Methods:**

The daily outpatient attendance of AOM for childhood (from 6 months to 12 years) was compared in the tertiary hospital in Shanghai during pre-COVID-19 and COVID-19 year.

**Results:**

A total of 24,543 AOM cases were included from 2015 to 2020. When age was taken into account, children in kindergarten (aged 4–6) constitute 66.2% (16,236/24,543) of all case, followed by primary school students (6,441/24,543, 26.2%) and preschoolers <3 years old (1,866/24,543, 7.6%). There was an estimated 63.6% (54.32–70.36%) reduction in the daily outpatient attendance of AOM associated with the introduction of social distancing in 2020 (COVID-19 year). The epidemic trend of AOM in 2015–2019 was characterized by seasonal fluctuations, with highest incidence in December (18.8 ± 0.5%) and lower in February (4.5 ± 0.2%), June (3.7 ± 0.7%) and August (3.5 ± 0.5%). And distribution characteristics of different ages in COVID-19 period broadly in line with that in non-pandemic period.

**Conclusion:**

Seasonal fluctuation in the prevalence of AOM was observed in pre-COVID-19 period (2015–2019), with a peak in winter and a nadir in summer. The >50% drop of outpatient attendance of AOM in 2020 (COVID-19 year) suggest that social distancing, mask effects and good hand hygiene can significantly reduce the incidence of AOM, which provides a preventive and therapeutic point of view for AOM.

## 1. Introduction

Acute otitis media (AOM) is a major worldwide healthcare problem in children, and more than 80% of children developed AOM more than once before 3 years old (1–4). The World Health Organization estimates that AOM induces a series of complications and sequelae, such as chronic suppurative otitis, which is a major cause of hearing loss in many developing countries (5, 6).

AOM presents with acute symptoms (<48 h), such as ear pain or erythema, associated with mild bulging of the tympanic membrane. Young children are at increasing risk of AOM, attributing to the immature morphological structure of the eustachian tube, naive immune function, and especially, high incidence of upper respiratory tract infection (7–9). Epidemiological evidence indicates that respiratory tract infection is the most important risk factor for AOM in children. The prevalence of AOM in children with respiratory infections usually ranges from 10 to 50% (3, 10, 11). It was corroborated that individual and social isolation in the Corona Virus Disease 2019 (COVID-19) pandemic period strictly hindered acute upper respiratory infections (12, 13). However, it is not clear that the prevalence of AOM descends by similar amounts, especially in children.

Therefore, in this study, we aimed to evaluate the prevalence of AOM in the Non-pandemic & COVID-19 Pandemic Period (2015–2020) and to explore additional, unintended benefits of social isolation, especially pandemic-related restriction on the natural history of AOM in children.

## 2. Methods

### 2.1. Participants

Daily AOM outpatient records from January 2015 to December 2020 were validated and acquired from a tertiary hospital in Shanghai. The change in daily outpatient attendance for AOM and time-series analyses were estimated following both non-pandemic and COVID-19 period.

Patients aging from 6 months to 12 years with a history of rapid onset (<48 h), presence of middle ear effusion, signs of middle ear infection were included in our study. Recurrent AOM was also included in our study. Electro-otoscopic evidence of eardrum perforation, tympanosclerosis, or cholesteatoma was excluded. And chronic otitis media with effusion was also excluded in this study.

All children underwent electro-otoscopy after removing earwax, pure tone audiometry and tympanometry were examined if necessary. The main demographic data and date of hospital visits were recorded in our study. Patients were divided into three groups by age with cut-off values 3 and 7, which categorized them into preschoolers (<3 years old), kindergarten children (4–6 years old) and primary school students (7–12 years old).

### 2.2. Ethical considerations

All procedures in studies were in accordance with the ethical standards of the World Medical Association's Declaration of Helsinki and its later amendments or comparable ethical standards. Ethical approval and patient consent were not required for this review. All data were handled according to Good Clinical Practice (GCP) guidelines.

### 2.3. Statistical analysis

Perform descriptive statistics on the occurrence of adverse reactions. Quantitative data will be described by mean ± standard deviation, or median (interquartile range), qualitative data are described by frequency and frequency. Analyses of Variance (ANOVA) were used to determine the significance of differences among groups on sex and age. All analyses were performed using R (version 3.5). The significance level was set as *p* < 0.05.

## 3. Results

### 3.1. Characteristics of patients

**Figure 1:**
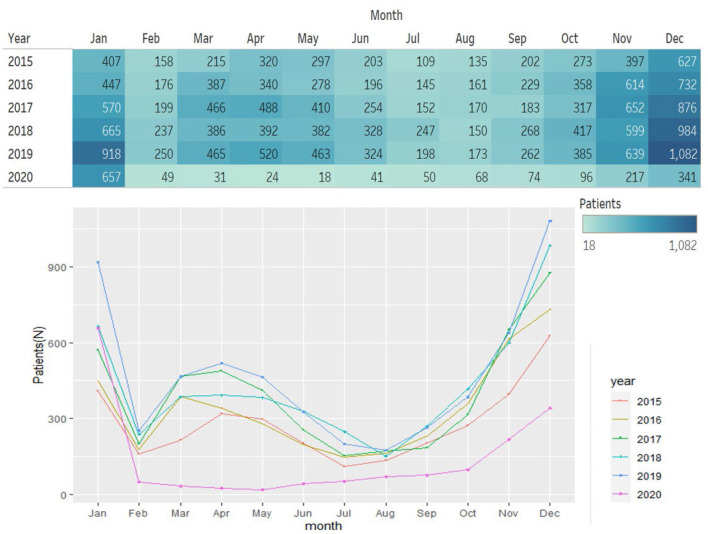
Number of AOM outpatients per month in 2015–2020.

The main demographic data and clinical characteristics of the outpatients are summarized in Table 1. A total of 24,543 AOM cases were included in the study. A higher prevalence of males (55%, *n* = 13,529) than females (45%, *n* = 11,014) was observed, with no statistical differences. Among the three typical age groups, children in kindergarten (aged 4–6) constitute 66.2% (16,236/24,543) of all case, followed by primary school students (6,441/24,543, 26.2%) and preschoolers <3 years old (1,866/24,543, 7.6%). This clear age distribution was observed every year from 2015 to 2020 (Figure 1).

**Table 1:** Characteristics of the study participants.

		**2015** **(*n* = 3,343)**	**2016** **(*n* = 4,063)**	**2017** **(*n* = 4,737)**	**2018** **(*n* = 5,055)**	**2019** **(*n* = 5,679)**	**2020** **(*n* = 1,666)**	**Overall** **(*n* = 24,543)**	***p***
Sex [N (%)]	Male	1,878 (56.2)	2,217 (54.6)	2,611 (55.1)	2,808 (55.5)	3,110 (54.8)	905 (54.3)	13,529 (55.1)	0.683
	Female	1,465 (43.8)	1,846 (45.4)	2,126 (44.9)	2,247 (44.5)	2,569 (45.2)	761 (45.7)	11,014 (44.9)	
Age [*N* (%)]	<3	281 (8.4)	291 (7.2)	378 (8.0)	401 (7.9)	392 (6.9)	123 (7.4)	1,866 (7.6)	<0.001
	4–6	2,145 (64.2)	2,752 (67.7)	3,173 (67.0)	3,390 (67.1)	3,699 (65.1)	1,077 (64.6)	16,236 (66.2)	
	≥7	917 (27.4)	1,020 (25.1)	1,186 (25.0)	1,264 (25.0)	1,588 (28.0)	466 (28.0)	6,441 (26.2)	

### 3.2. Monthly epidemic trend of AOM outpatients in non-pandemic and COVID-19 pandemic period (2015–2020)

**Figure 2:**
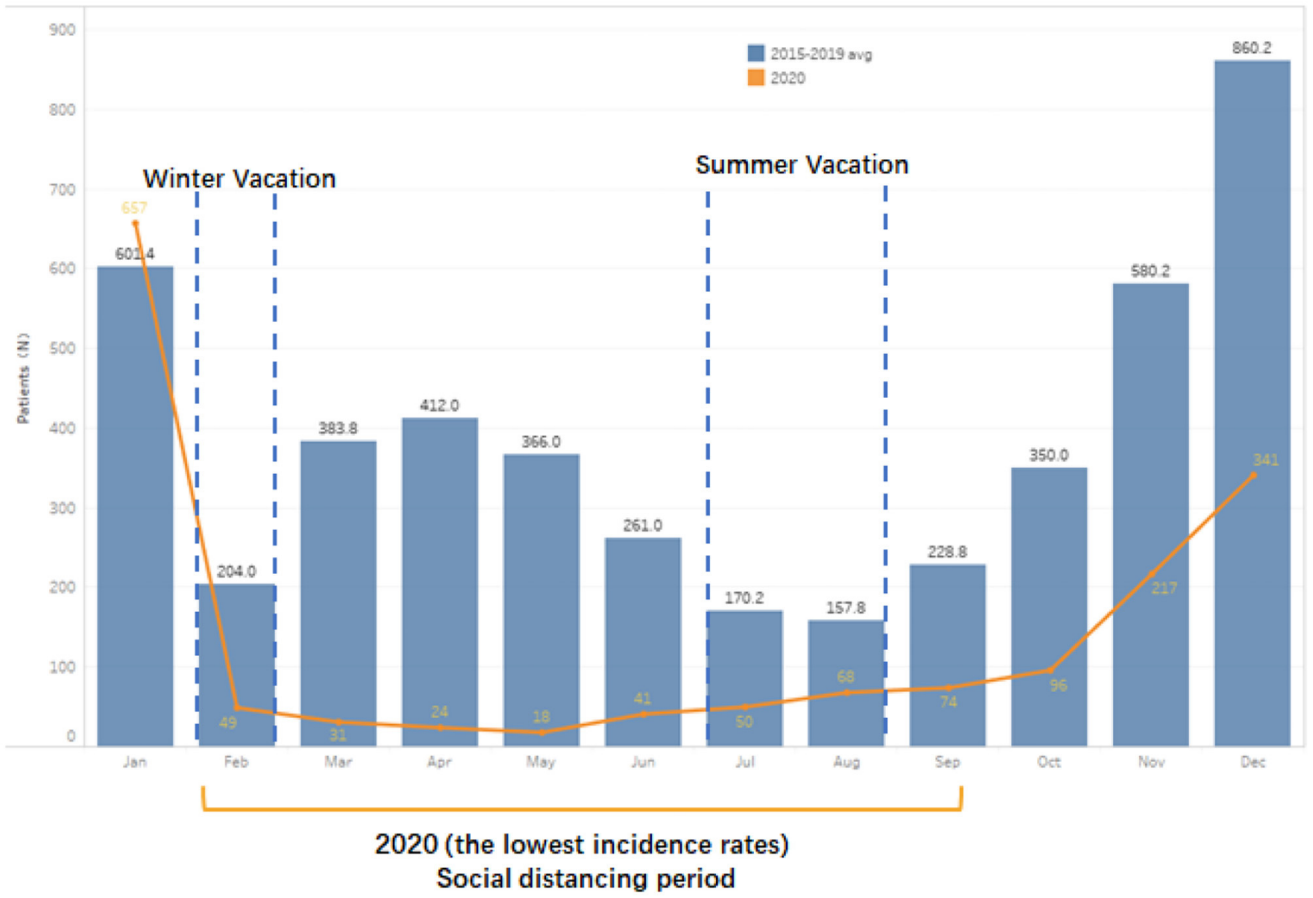
Number of AOM outpatients monthly in Non-pandemic and COVID-19 Pandemic Period (2015–2020).

Generally, the epidemic trend of AOM outpatients in 2015–2019 was basically the same with seasonal fluctuations. In terms of months, the outpatient rate of AOM was higher in November (12.8 ± 1.6%), December (18.8 ± 0.5%), and January (12.9 ± 2.0%); while AOM outpatient records were relatively lower in February (4.5 ± 0.2%), June (3.7 ± 0.7%) and August (3.5 ± 0.5%) (Figures 1, 2). Social distancing of COVID-19 caused a dramatic decline in AOM outpatient attendance (63.6%, 95%CI, 54.32–70.36%). The number of AOM outpatient monthly visits sustained lower in 2020, especially after January. The greatest reductions were seen in May, which reduced by 90%. Since October, the epidemic trend of AOM outpatients began to rise slowly (Figures 1, 2).

**Figure 3:**
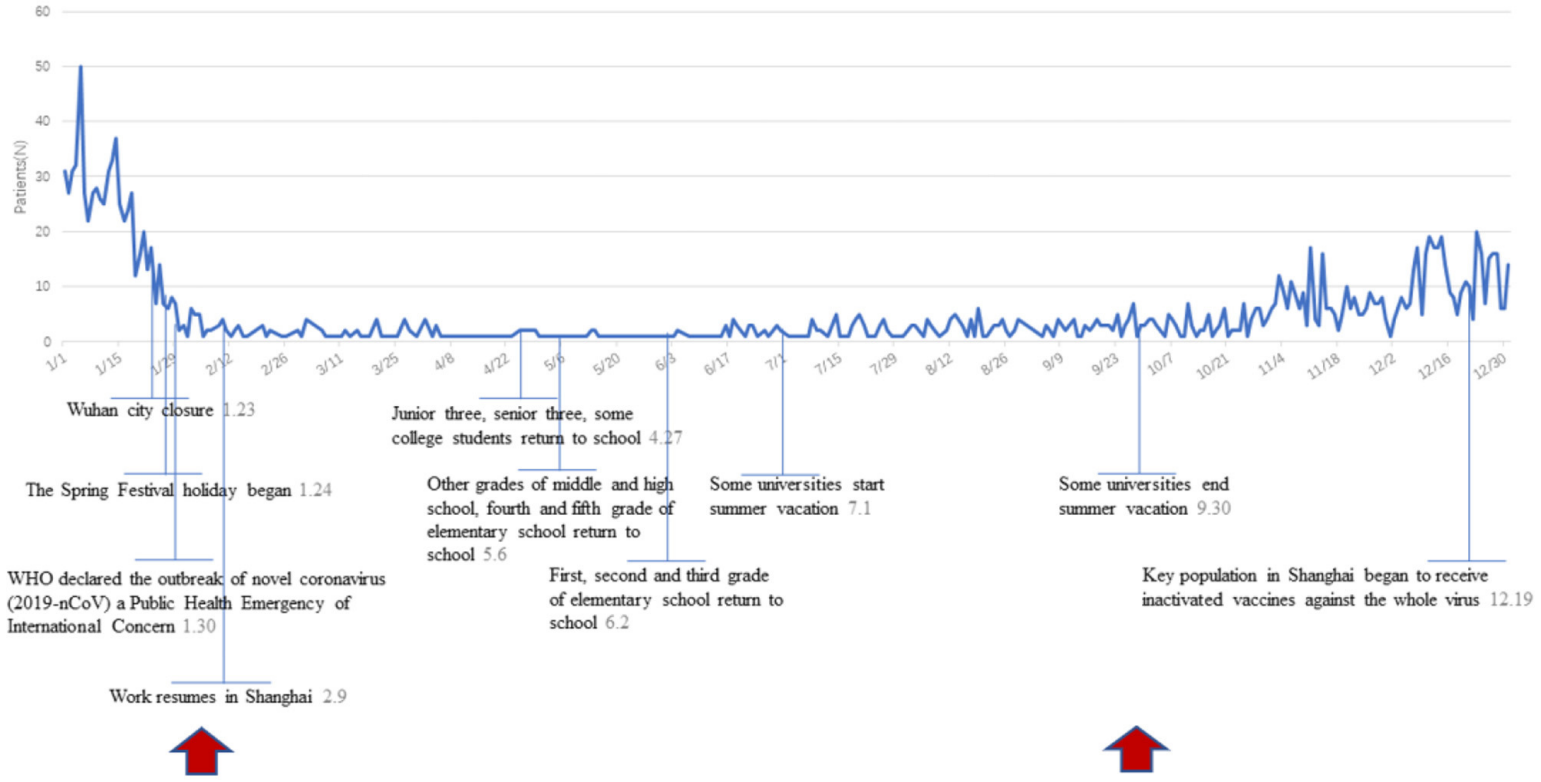
Daily AOM outpatients following time series chart of COVID-19 events.

The main events and daily epidemic trend of AOM outpatients in the COVID-19 pandemic period was showed in Figure 3. Chinese government enforced a lockdown in Wuhan in late January, and the World Health Organization declared the outbreak of novel coronavirus a Public Health Emergency of International Concern shortly afterward. After that, the number of outpatients plummeted, significantly lower than the average level throughout the year.

### 3.3. Age-dependent analysis of AOM epidemiological trends in 2015–2020

**Figure 4:**
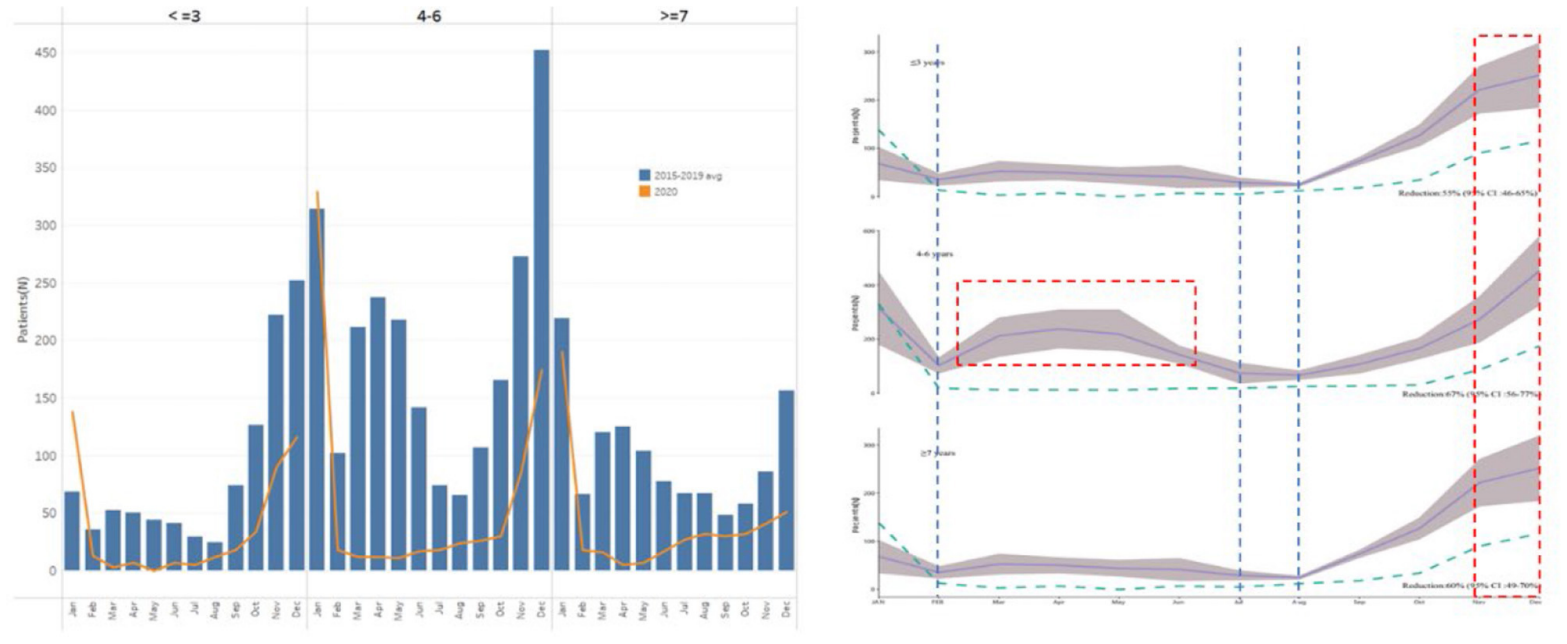
Age-dependent analysis of AOM epidemiological trends in 2015–2020. The higher outpatient rates of AOM were marked in red line; while lower AOM outpatient records were marked in blue line.

Our survey found that children aged from 4 to 6 years account for more than half of the AOM cases. The average AOM epidemic trend during 2015 to 2020 for different age groups was illustrated (Figure 4). There was a significant difference of AOM occurrences in different seasons among different ages (*p* < 0.01). For children <3 years old, autumn and winter (September, October, November, December) were the seasons of high AOM occurrences. For children in kindergarten aged 4–6 years, they should be cautious of suffering from AOM throughout the year except February, July, and August. The seasonal or monthly AOM epidemic trends became less pronounced for primary school students (aged from 7 to 12) although it slightly fluctuated in winter.

The age distribution of AOM was not affected significantly by the epidemic situation in 2020. In the COVID-19 pandemic period, the seasonal effect of AOM was similar in preschoolers and children in kindergarten, with remarkable increases in autumn and winter. Meanwhile, the number of AOM outpatients in primary school students gradually went up since May.

## 4. Discussion

The prevention and treatment of AOM is one of the top priorities in otolaryngology department. Recent studies revealed that viral infection of the respiratory tract, attendance of day-care centers and exposures to smoke have increased the likelihood of developing AOM (4). It was reported that a lower incidence of otitis media with effusion was found in Italy, followed by restrictive anti-contagion measures (14, 15). In relative terms, there is a higher overall incidence of AOM and it is more closely related with respiratory tract infection in children, such as COVID-19. So far, few studies focus on additional assessment of the effects of social distancing on the incidence of AOM. Patel et al. reported that otitis media was felt to be decreased by 85.7% due to strictest and average restrictions during the majority of the pandemic (16). We thus attempt to investigate more impact of activities parallel to daily living, including the COVID-19 lockdown, on the number of admissions for ENT-related AOM at a large pediatric ENT emergency department, located in China.

First, we speculated that fewer outdoor social activities for children play a critical role in reducing the incidence of AOM. It's well-known that summer vacation is in July and August and winter vacation in February in China. And in our observational survey, a lower prevalence of AOM in the 3 months. It is worth noting that AOM prevalence dropped sharply in February (<300 children) every year from 2015 to 2019 in our data. However, outpatient visits for AOM in January and March (before and after February) nearly treble or double that in February. Seasonally adjusted, the significant reduction of social gathering among children results in a lower prevalence of AOM in winter and summer holidays.

Second, this research also allowed analysis into AOM prevalence across different age groups, and found that children aged from 4 to 6 years had the highest incidence of the disease. Importantly, the incidence of AOM didn't simply increase or decrease with age, and was involved in social and demographic factors. It was families that children spent most of their time with before 3 years old. Children aged 4–6 started school life gradually, along with more crowd gathering activities, and thus the AOM prevalence increased greatly. After age 7, children are less likely to get AOM, indicating changes in the fully-grown morphological structure of the eustachian tube and progressive maturation of the immune system (17–19). Seasonal variations of AOM prevalence in children at different ages were also diverse. Even during non-epidemic periods, the incidence of AOM in school-age children was closely related to the semesters, which decreased significantly in winter and summer vacation. Therefore, there was a positive effect of social distancing on the AOM prevalence for school-age children. On the other hand, the incidence of AOM in preschoolers was more correlated with season variations, but not semesters.

Third, climate change along with seasonality in the area is recognized as an important factor in the incidence of AOM (20). In agreement with previous studies, seasonal fluctuation in the prevalence of AOM was observed in 2015–2019, with a peak in winter and a nadir in the summer. This may be related to the high incidence of upper respiratory tract infections in autumn and winter (21). In autumn and winter, the influenza virus leads to a significant increase in upper respiratory tract infections and therefore results in a high incidence of AOM (20–22).

During the COVID-19 pandemic period, patients' choice of hospitals and willingness to see doctors were affected, so there would be small impacts on the statistical results. Although AOM is emergency, the choice of treatment is less affected by the influence, it should be explained in the discussion section.

In addition, social distancing, mask effects and good hand hygiene may account for the decrease in the prevalence of AOM in the COVID-19 year. A nationwide lockdown was imposed by the Chinese government in 2020 to prevent the spread of COVID-19. Besides the reasons above, patients' choice of hospitals and willingness to see doctors would also have some impacts on the statistical results during the COVID-19 pandemic period. The compliance with hygienic-behavioral rules and restriction of unnecessary interpersonal contacts contributed to controlling the spread of diseases related to upper respiratory tract infection, besides COVID-19 (23–25). Social distancing, due to the outbreak of COVID-19, also caused a dramatic decline of AOM outpatient attendance, especially from February to August, when kindergartens and primary schools closed for home-based online teaching. After September, we have resumed our normal social and teaching activities in China, and the incidence of AOM increased significantly compared with the previous few months. But generally, the AOM outpatient attendance has dropped by more than 50% compared with that in the same period of previous years. It suggests that isolation effects caused by wearing masks could avoid or slow the spread of disease to some extent, which contributed to the major reduction of AOM cases.

Despite the importance of social distancing in preventing AOM prevalence, there are still several limitations in our study. For convenience, narratives are constructed from limited data in a single audiologic center. Importantly, our study is a retrospective and hospital-based analysis so possible bias and confounding may exist when some patients postponed or refused to go to the hospital due to the initial outbreak of COVID-19.

## 5. Conclusion

Based on our study, we presume that the influencing factors of AOM prevalence are mainly: interpersonal distance, mask effects, seasonal variation, age factors, etc. Social distancing could effectively reduce the likelihood of catching AOM since data from the comparison between non-pandemic and COVID-19 periods indicated that AOM might spread into susceptible population by close contact. Importantly, lower AOM attack rates during social distancing underscores the role of non-pharmaceutical interventions and reductions of direct person-to-person contact, which provides a preventive and therapeutic point of view for AOM.

It is a report focusing on the effects of social distancing on AOM prevalence in children from both non-pandemic and COVID-19 period in China. The present study highlighted that there was a lower prevalence of AOM among the pediatric patients during both vacations of non-pandemic period and rigorous social distancing of COVID-19 period. The phenomenon of AOM periodic fluctuation in this study suggested that good classroom ventilation, hand hygiene education and isolation with upper respiratory tract infection may contribute to the reduction of AOM and its cross-infection in schools. It should be emphasized, AOM epidemic trends peak in spring only in school-age children, which has a notable difference from other children and requires in-depth research on the reasons.

A rigorous prospective, population-based study are needed to identify whether keeping interpersonal distance and wearing masks could be a viable alternative in the treatment of AOM in children, especially during the epidemic of upper respiratory tract infection. And further investigation is also need to explore the role of influenza virus in the epidemic trends of AOM in the children of different age.

## Data availability statement

The raw data supporting the conclusions of this article will be made available by the authors, without undue reservation.

## Ethics statement

Ethical review and approval was not required for the study on human participants in accordance with the local legislation and institutional requirements. Written informed consent from the participants' legal guardian/next of kin was not required to participate in this study in accordance with the national legislation and the institutional requirements.

## Author contributions

QL: substantial contributions to conceptualization, supervision, validation, review and editing of the work. HY: substantial contributions to the design of the work, original draft, visualization, data curation, project administration, and investigation. DG: data curation, formal analysis, and methodology and software for the work. FY: formal analysis and methodology and software. All authors contributed to the article and approved the submitted version.
